# The Lanostane Triterpenoids in *Poria cocos* Play Beneficial Roles in Immunoregulatory Activity

**DOI:** 10.3390/life11020111

**Published:** 2021-02-01

**Authors:** Chien-Liang Chao, Hsin-Wen Huang, Muh-Hwan Su, Hang-Ching Lin, Wen-Mein Wu

**Affiliations:** 1 Sinphar Pharmaceutical Co., Ltd., Sinphar group, Yilan 269, Taiwan; chaokmc@gmail.com (C.-L.C.); HWHuang@sinphar.com.tw (H.-W.H.); MHSu@syncorebio.com (M.-H.S.); 2 School of Pharmacy, National Defense Medical Center, Taipei 114, Taiwan; 3 Department of Nutritional Science, Fu-Jen Catholic University, Hsinchuang 24205, Taiwan

**Keywords:** *Poria cocos*, lanostane triterpenoids, immunotoxicity, Th1/Th2, immunoregulation

## Abstract

*Poria cocos* (Schwein) F.A. Wolf (syn. *Wolfiporia cocos*) dried sclerotium, called fuling, is an edible, saprophytic fungus commonly used as a tonic and anti-aging traditional Chinese medicine. It is traditionally used in combination with other traditional Chinese medicines to enhance immunity. This study showed that *P. cocos* extract (Lipucan^®^) containing lanostane triterpenoids has no immunotoxicity and enhances non-specific (innate) immunity though activating natural killer cells and promotes interferon γ (IFN-γ) secretion by Type 1 T-helper (Th1) cells immune response. In addition, *P. cocos* extract significantly decreased interleukin (IL-4 and IL-5) secretion by Type 2 T-helper (Th2) cells immune response, which are related to the allergy response. The purified lanostane triterpenoids were first identified as active ingredients of *P. cocos* with enhanced non-specific immunity by promoting interferon γ (IFN-γ) secretion in a preliminary study. Our findings support that the *P. cocos* extract plays beneficial roles in immunoregulatory activity.

## 1. Introduction

Virus infections such as respiratory virus (including influenza virus, rhinovirus, adenovirus and coronavirus), herpes and human immunodeficiency virus (HIV) seriously threaten human health. These highly contagious respiratory viruses infect the world’s population, become pandemic and cause a lot of deaths. This threat has evolved into a personal health issue and also into international economic, safety and social issues [1]. Vaccination is currently the primary means of controlling the spread of influenza virus infections. However, due to the virus’s notorious ability to mutate, new vaccines must be developed every year. There is an urgent need to develop effective antiviral drugs or therapeutic approaches. Unfortunately, many years and hundreds of millions of dollars are necessary to develop new medicines, often too late to fight a sudden virus epidemic. Recent reports indicated that people over the age of 50 are mainly infected with respiratory virus [2,3]. Aging is one reason closely related to defects in the immune system, including cell function and number [4,5,6]. Self-care is an important approach used in most countries to reduce personal medical expenditures and social health care burden [7,8]. The development of immunity enhancers to increase the host’s resistance to viral infection and improve the host’s adaptability is an important approach that has received lots of attention recently [9,10].

The human body’s first line of defense against pathogens includes physiological barriers such as the skin, subcutaneous tissue and mucosa. The second line of defense is the immune system, composed of immune organs, immune cells and immune molecules. The immune system is divided into innate immunity and adaptive immunity [11]. The innate immune system is a non-specific immune system. It can distinguish between itself and non-self without repeated exposure to pathogens such as bacterium and viruses. Because of its non-specific characteristics, it has a broad ability to fight multiple infections [12]. Natural killer (NK) cells and interferon (IFN) are the key antiviral components of the innate immune system in host defense against respiratory viral infection [13]. NK cells have the ability to rapidly kill cells infected by viruses. Moreover, NK cells also trigger other immune cells, Type 1 T-helper (Th1) cells, by releasing IFN-γ [14,15]. Interferons have the ability to interfere with viral replication and can be divided into three types including interferons I (IFN-α, IFN-β), II (IFN-γ), and III (IFN-λ) [16,17]. Virus replication is an important basic stage of virus life. Th1 cells play an essential role in defending against virus infection [18]. On the other hand, Type 2 T-helper (Th2) cells mainly secrete interleukins (ILs) including IL-4, IL-5, and promote B cells to secrete immunoglobulin E (IgE) antibodies to promote humoral immunity and induce allergy response [19].

Fuling (the dried sclerotium of *Poria cocos* (Schwein.) F.A. Wolf (syn. *Wolfiporia cocos*)), a well-known tonic and anti-aging traditional Chinese medicine, has been widely used as a sedative and diuretic for more than two thousand years [20]. *P. cocos* has been demonstrated to have anti-inflammatory, anti-tumor, anti-hyperglycemic, sedative, and anti-aging functions with lanostane triterpenoids identified as the active components [21,22,23,24,25,26,27,28,29,30]. In addition, the ethyl acetate fraction and crude polysaccharide fraction of *P. cocos* have been shown to enhance immunity in animal models based on the serum hemolysis content test, phagocytic effect of mononuclear macrophages and the level of lymphocyte transformation. Lanostane triterpenoids were considered major components in the ethyl acetate fraction according to HPLC analysis [31]. However, it is still unclear that the innate and adaptive immunity have effects on NK cells activity, IFN, immune cells or cytokines of *P. cocos* and the active compounds clarification. This study investigated the immune system effect of *P. cocos* extract, a patented lanostane triterpenoids content consistency product, using animal models. The active components were identified first.

## 2. Materials and Methods

### 2.1. Plant Materials

**Figure 1:**
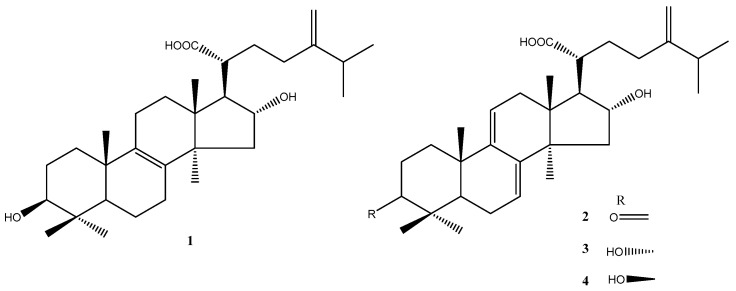
The chemical structures of lanostane triterpenoids **1**–**4** isolated from *P. cocos*.

The dried selerotium of *P. cocos* (Schwein.) F.A. Wolf was extracted using 75% ethanol to obtain *P. cocos* extract (Lipucan^®^). This extract is manufactured by Sinphar Tian-Li Pharmaceutical Co., Ltd., Hangzhou Sinphar Group, China, and developed by Sinphar R&D Center, Taiwan. The extract contains four major lanostane triterpenoids (Figure 1, compounds **1**–**4**) analyzed using ultra-performance liquid chromatography (UPLC) [32]. The four major lanostane triterpenoids content was 6.2%. A commercial capsule (FL) that contains 27.0 mg of *P. cocos* extract was used to investigate the effect on immuneregulatory activity.

### 2.2. Isolation and Purification of Lanostane Triterpenoids from P. cocos

The dried *P. cocos* (10 kg) was extracted three times by refluxing with 75% ethanol for 3 h [24]. The concentrated extract was chromatographed on silica gel (70–230 mesh) using increasingly polar mixtures of CH_2_Cl_2_ and MeOH (CH_2_Cl_2_:MeOH, 97:3; CH_2_Cl_2_:MeOH, 96:4; CH_2_Cl_2_:MeOH, 90:10, and 100% MeOH). According to the thin-layer chromatography (TLC), four fractions (Fr.1–Fr.4) were collected for further separation. The Fr.1–Fr.3 were subjected to preparative high-performance liquid chromatography (HPLC) (Waters Prep 150 LC system, Milford, MA, USA) on a Waters XBridge RP-18 column (250 mm × 19 mm, 5 μm, Milford, MA, USA) using 80% methanol as the mobile phase system. The flow rate was 18 mL/min. Four major peaks of interest were selectively collected. The fractions containing the targeted compounds were further condensed into dryness and produced tumulosic acid (**1**) (120.1 mg), polyporenic acid C (**2**) (16.0 mg), 3-epi-dehydrotumulosic acid (**3**) (12.1 mg), and dehydrotumulosic acid (**4**) (6.8 mg), respectively. Their structures were elucidated by NMR (nuclear magnetic resonance) spectroscopy analysis and electrospray ionization mass spectrometer (ESI-MS) and by comparison with literature data [32].

### 2.3. Preliminary Animal Study by Lanostane Triterpenoid Compounds *(****1***–***3****)* for IFN-γ Analysis Study

For this study, the purified lanostane triterpenoid compounds, including tumulosic acid (**1**), polyporenic acid C (**2**), and 3-epidehydrotumulosic acid (**3**), were prepared for the preliminary study using BALB/c (a mouse strain of albino mice) male mice. After 4 days’ oral administration of compounds **1**–**3** and sterile distilled water (control group) (1 mL/mice), mice were sacrificed at the fifth day, and spleen cells were collected. The fresh spleen was transferred to a culture plate containing 10 mL of Roswell Park Memorial Institute (RPMI)-1640 culture medium. The spleen was then ground over a fine mesh to release the spleen cells. The spleen cells suspended in the medium were then transferred to a 50 mL conical centrifuge tube and centrifuged at 1300 rpm for 10 min. The supernatant was discarded and the pellet was resuspended in 1 mL of cold red blood cells (RBC) lysing buffer containing EDTA-NH_4_Cl. The cells were incubated at room temperature for 10 min and then washed three times with culture medium by centrifugation. The spleen cell suspension was then cultured in a 24-well plate at a density of 1 × 10^6^ cells/mL in a medium containing RPMI 1640 supplemented with 10% fetal bovine serum (FBS), 2 mM l-glutamine, antibiotics and 1 μg/mL concanavalin A (ConA) at 37 °C for 3 days. Culture spleen cell supernatants were collected. The IFN-γ concentrations were measured using a Enzyme-linked immunosorbent assay (ELISA) kit (R&D Systems, Minneapolis, MN, USA).

### 2.4. Animal Model and Experimental Schedule

Female BALB/c mice were purchased from the National Taiwan University Animal Center. Animals were housed in individually ventilated cages for Specific-Pathogens-Free at 22 ± 2 °C, with temperature and humidity at 40–60% with a 12 h/12 h on light/dark cycle and free access to food and water. After a one-week acclimatization, the mice were randomly grouped according to body weight and used for experiments. Four different doses, 26 mg/kg (FL200), 52 mg/kg (FL400), 104 mg/kg (FL800), 156 mg/kg (FL1200), were respectively dissolved in sterile distilled water (0.4 mL) and orally administered for five consecutive days a week for 9 weeks. The control group was fed with sterile distilled water (0.4 mL). The mice were injected with ovalbumin (OVA)-specific antigen intraperitoneally on the third, fifth, and seventh week. OVA is a chicken egg white allergen found mainly in egg whites. It is usually used to induce allergies in experimental animal models. The spleen cells were collected for further study. The mice were sacrificed using carbon dioxide euthanasia after 9 weeks of experimentation. The approval number for this study by the institutional animal care and use committee (IACUC) is A9647.

### 2.5. Collection of Spleen Samples

The spleen, a dark red elongated organ in the upper left side of the mice abdomen, was collected. After the connective tissue was carefully removed using small scissors and forceps, spleens were placed in cell culture medium (RPMI 1640 medium with 10% fetal bovine serum (HyClone)). After weighing, spleen samples were ground using a clean and sterile 5 mL syringe pusher. The spleen cell suspension was aspirated into a new 15 mL centrifuge tube with a 3 mL sterile single-package plastic dropper. The suspended cells were collected after 5 min precipitation. The suspended cells were centrifuged at 1500 rpm for 7 min and the supernatant discarded to obtain the cell pallets. Then, 1 mL RBC lysis buffer was added to remove red blood cells for 1 min. Nine mL 10% fetal bovine serum was quickly added to the culture medium, the centrifugation step repeated, and the supernatant discarded. To avoid spleen cell integrity damage due to RBC lysis buffer, the sample was washed three times with Hank’s Balanced Salt Solution (HBSS) buffer solution. The spleen cells were then suspended with 10% fetal bovine serum culture medium for analysis and experiments.

### 2.6. Non-Specific Immune Response

#### 2.6.1. Spleen Cell Surface Marker Analysis

Immune cell analysis was performed using fluorescent monoclonal antibodies that specifically bind to various kinds of immune cells using fluorescent flow cytometer. The flow cytometry (Epics XL-MCL Beckman Coulter, Brea, CA, USA) is used to calculate the proportion of a specific immune cell such as major histocompatibility complexes type II (MHC II), CD4^+^ T cell, CD8^+^ T cell, NK cells and macrophages.

#### 2.6.2. Natural Killer Cells Activity Analysis

The YAC-1 (a cell line is sensitive to the action of NK cells activity) mouse lymphoma cell line (ATCC) was used as the NK cells target in this experiment. When BALB/c female mice spleen cells were co-cultured with YAC-1 cells in the same dish, natural killer cells will kill YAC-1 cells. After 3 h of cytotoxicity reaction, the killed YAC-1 cells were stained with a dye (LIVE/DEAD Cell-Mediated Cytotoxicity kit, Molecular Probes, L-7010). Therefore, the flow cytometry was used to detect and analyze the fluorescence intensity using WinMDI 2.8 software (Purdue University Cytometry Laboratories, West Lafayette, IN, USA). The effector cell (E) to the target cell (T) ratio is 100:1 and 200:1 (The effector cel1 is spleen cells and the target cell is YAC-1 cells).

#### 2.6.3. Cytokines Secretion Using Spleen Cells Analysis

After stimulating the spleen cells with ConA (concentration 2.5 μg/mL) for 48 h, the cell culture supernatant was collected and stored at −20 °C for the cytokines analysis using a OptEIA mouse IL-5 ELISA kit (Pharmigen, 555236, Franklin Lake, NJ, USA) and a DouSet mouse IFN-γ ELISA kit (R&D Systems, DY485, Minneapolis, MN, USA). Coating buffer (pH: 9.6) was prepared to contain the appropriate amount of anti-mice cytokine antibodies (IL-5 and IFN-γ) on a 96-well plate (Nunc-Immuno plate, MaxiSorp, Thermo Scientific, Roskilde, Denmark). After standing at 4 °C overnight, the unbound antibodies were rinsed with the Phosphate Buffered Saline with Tween^®^ 20 (PBST) buffer, and 200 μL/well of blocking buffer (1% BSA in PBS) was then added. After 2 h at room temperature, the sample was rinsed with PBST buffer, and 100 μL/well of cell culture supernatant or recombinant cytokine standard added to the sample. After 4 °C overnight, the sample was rinsed with PBST buffer. The appropriate concentration of linked biotin (biotin) anti-cytokine secondary antibody (100 μL/well) was then added. After 2 h at room temperature, the sample was rinsed with PBST buffer. Avidin-peroxidase (100 μL/well) (Sigma, St. Louis, MO, USA) was then added. After 1 hour at room temperature, Tetramethylbenzidine (TMB) (R&D Systems, Minneapolis, MN, USA) substrate was added for 5 min of color reaction, and 50 μL 2.5% H_2_SO_4_ was then added to stop the color reaction. The absorbance was measured at 450 nm.

### 2.7. Specific Immune Response by Ovalbumin (OVA)-Induced Mice

BALB/c female mice were injected intraperitoneally with OVA as the antigen, and CFA (Complete Freund’s Adjuvant) as the adjuvant. The spleen cell culture conditions were the same as above. Cytokines (IL-4) were analyzed by ELISA.

### 2.8. Statistical Analyses

Data were reported as mean (SD), and analyzed using one-way ANOVA. Values were considered statistically significant at *p* < 0.05. Dunnett’s test was used to identify the differences between groups.

## 3. Results

### 3.1. Isolation and Identification of Four Lanostane Triterpenoid Compounds ***1***–***4*** of P. cocos

The dried *P. cocos* (10 kg) was extracted thrice by refluxing with 75% ethanol for 3 h. The concentrated extract was chromatographed on silica gel and C_18_ column to furnish four major lanostane triterpenoid compounds: tumulosic acid (**1**), polyporenic acid C (**2**), 3-epi-dehydrotumulosic acid (**3**), and dehydrotumulosic acid (**4**), respectively (Figure 1). Their structures were elucidated by NMR spectroscopy (Table S1) and ESI-MS analysis (Figures S2, S4, S6, and S8) and comparison with literature data [32,33]. The UPLC chromatograms of **1**–**4** were showed in Figures S1, S3, S5, and S7.

### 3.2. Preliminary Study in BALB/c Male Mice by Lanostane Triterpenoid Compounds ***1***–***3***

The Spleen cells isolated from the mice treated with lanostane triterpenoid compounds (**1**–**3**) were cultured for five days in the presence of ConA. The amount of IFN-γ secreted by the spleen T lymphocytes was measured. After the mice were fed with 2.5 mg/kg/day or higher dose of compound **1**, 5 and 10 mg/kg/day of **2**, and 20 mg/kg/day of **3**, respectively, the IFN-γ secreted by ConA-stimulated splenic T cells was significantly augmented (Table 1 and Table 2). The preliminary study shows that lanostane **1**–**3** increased IFN-γ secretion by spleen cells stimulated by ConA.

### 3.3. Safety Assessment from Animal Study of P. cocos Extract

Table 3 and Table 4 indicate that *P. cocos* extract did not affect body weight and spleen weight. Table 5 also displays that the immune cells such as total T cells, total B cells, MHC II (major histocompatibility complex type II), CD4^+^ T cells, CD8^+^ T cells, NK cells, and macrophages in the *P. cocos* extract group had no significant difference compared with the control group. Based on the above results, it should be assessed that there should be no risk of immunotoxicity during the *P. cocos* extract feeding experiment. 

### 3.4. Non-Specific Immune Response Assessment

Nature Killer Cells (NK cells) play a pivotal role in non-specific immunity. Table 6 shows that the FL400 group significantly induced an increase in NK cells activity compared with the FL200 group. It is a tendency of increased effect of NK cells activity of *P. cocos* extract. Cytokines are chemicals including interleukins (IL) and interferon (IFN) that regulate immune response or cell growth [34]. We analyzed IFN-γ (Th1 immune response) concentration of ConA and LPS-induced spleen cells and IL-5 (Th2 immune response) concentration of ConA-induced spleen cells isolated from mice treated with FL200, FL400, FL800, and FL1200 (Table 7 and Table 8). The FL800 and FL1200 groups significantly stimulated IFN-γ production in mice spleen cells in the presence of ConA (Table 7). Table 7 also shows that the IL-5 mice spleen cell concentration isolated from mice treated with FL200, FL400, FL800, and FL1200 had significantly decreased effect in a dose-dependent manner. In addition, the FL400, FL800, and FL1200 group also significantly stimulated IFN-γ production in mice spleen cells in the presence of LPS (Table 8).

### 3.5. OVA-Induced Specific Immune Response Assessment

In this study, there was a tendency to suppress IL-4 secretion in *P. cocos* extract treatment (Table 9). Especially, IL-4 (Th2 immune response) was significantly decreased in the FL1200 group in OVA-induced mice.

## 4. Discussion

The human immune system is responsible for fighting foreign pathogens to protect health. Insufficient immunity usually makes the body susceptible to infection and therefore requires sufficient immunity, but it also requires strict regulatory mechanisms to avoid excessive collateral damage. Maintaining immune balance is the most important immune system task [35]. Lots of evidence indicated that immune balance is highly correlated with the Th1/Th2 cell response [36,37]. Stress and aging may cause Th1/Th2 to lose balance and tilt toward Th2, which may cause infections and allergic diseases [38,39,40]. This search for effective balance immunity is in urgent need. In addition, it is gratifying that the knowledge gained from decades of accumulated scientific research on the human immune system and its response to infectious diseases helps to provide information on therapeutic research and development, and also has preventive strategies for the spread of virus outbreaks [41,42]. Many previous studies on the pharmacological study of *P. cocos* are biased towards *P. cocos* protein [43,44] or polysaccharide [45,46]. There are several studies on the efficacy of lanostane triterpenoids from *P. cocos* such as hypoglycemia [25], anti-cancer [24], and sedative function [28]. Previous studies have shown that the ethyl acetate fraction of *P. cocos* contains the main component triterpenoids and has immune-enhancing activity [31]. However, no further follow-up in-depth research has been published. Our study evaluated the *P. cocos* immunity function in a well-established mice model including the purified lanostane triterpenoid compound preliminary screening study. We showed in this study that the *P. cocos* extract (Lipucan^®^) containing 6.2% of four lanostane triterpenoids plays multi-beneficial roles in immunoregulatory activity. 

A previous investigation reported that Human CD4^+^ T-helper (Th) cells including Th1 and Th2 subsets are defined by the cytokines they secrete [36]. Th1 cells mainly secrete IFN-γ; Th2 cells produce IL-4, IL-5, induce antibody production, and lead to allergic responses by increasing IgE production by B cells, and promote mast cell growth and eosinophil differentiation. It is well known that NK cells and IFN-γ play important roles in immune defense against virus infections. The innate immune system is very important to defend against viruses that initially invaded the body and activate subsequent adaptive immunity. NK cells are classified as non-specific (innate) immunity responsible for killing virus-infected cells [14]. IFN-γ inhibits virus life cycle and prevents virus replication. IFN-γ also regulates the immune response by activating non-specific cell-mediated immunity and stimulating specific immunity [17]. Based on the preliminary animal study, the main lanostane triterpenoid compounds of *P. cocos* extract, tumulosic acid (**1**), polyporenic acid C (**2**), and 3-epi-dehydrotumulosic acid (**3**), significantly stimulate IFN-γ secretion by spleen cells. Active *Poria cocos* extract component confirmation in this preliminary study will be of great significance to the quality control of product and further bioavailability and mechanism studies. Furthermore, we showed in this study that *P. cocos* extract significantly stimulates NK cells activity and IFN-γ secretion with no immunotoxic properties. These results demonstrated the significant stimulatory effects of *P. cocos* extract and the main lanostane triterpenoids **1**–**3** on Th1 immune response.

Moreover, our findings indicated that *P. cocos* extract suppressed the Th2 immune response by significant IL-5 inhibition (Table 7) on non-specific immune response model, and significant IL-4 (Table 9) inhibition on OVA-induced specific immune response model. IL-4 and IL-5 would lead to an allergic response. Allergies, also celled allergic diseases, are caused by an immune system being hypersensitive to substances in the environment, such as allergic asthma. The patient’s immune response tends to Th2. If Th1/Th2 in the body can be balanced, it should improve the allergy symptoms. This study proved that *P. cocos* extract can regulate Th1/Th2 immune response, may reduce the occurrence of allergic diseases and can be developed into a potential candidate with anti-allergic disease.

## 5. Conclusions

This study is the first to demonstrate the non-specific and specific immunity regulation of *P. cocos* extract action containing lanostane triterpenoids in mice. These immune responses include the activation of NK cells, increased IFN-γ secretion, and decreased IL-4 and IL-5. Our findings support that *P. cocos* extract with no immunotoxicity added to the diet or used alone will play beneficial immunoregulatoy roles in immunodeficiency improvement and the improvement of the ability to prevent infection and allergy responses. This is first confirmation that lanostane triterpenoids are effective ingredients and can be used as quality control ingredients to maintain the consistency of *P. cocos* extract efficacy.

## Figures and Tables

**Table 1:** Tumulosic acid (**1**) increased IFN-γ secreted by spleen T lymphocytes.

Components	Dosage (mg/kg/Day)	IFN-γ (pg/mL)
**1**	2.5	414.80 ± 31.3 *
	5	432.70 ± 50.22 *
	10	348.45 ± 72.56 *
	20	457.48 ± 57.60 **
**Control**		254.14 ± 55.68

Data were expressed as mean ± SEM (n = 10). Data were analyzed statistically using one-way ANOVA and Dunnett’s test. * *p* < 0.05 and ** *p* < 0.01 compared with the control group.

**Table 2:** Polyporenic acid C (**2**) and 3-epi-dehydrotumulosic acid (**3**) increased IFN-γ secreted by the spleen T lymphocytes.

Components	Dosage (mg/kg/Day)	IFN-γ (pg/mL)
**2**	5	917.07 ± 130.41 *
	10	449.74 ± 100.67 *
	20	176.20 ± 45.96
**3**	5	240.45 ± 107.83
	10	252.26 ± 103.76
	20	292.00 ± 77.77 *
**Control**		124.25 ± 28.15

Data were expressed as mean ± SEM (n = 6). Data were analyzed statistically using one-way ANOVA and Dunnett’s test. * *p* < 0.05 compared with the control group.

**Table 3:** Mice body weight records.

Group	Control	FL200	FL400	FL800	FL1200
Weeks	Body Weight (g)				
1	19.38 ± 0.95	19.37 ± 1.00	19.36 ± 1.02	19.38 ± 1.02	19.40 ± 1.07
4	20.75 ± 1.37	20.88 ± 1.09	21.11 ± 1.00	20.90 ± 1.15	20.50 ± 1.60
5	18.24 ± 1.14	18.38 ± 1.45	18.95 ± 1.06	18.66 ± 1.45	18.22 ± 1.35
6	20.24 ± 0.93	20.64 ± 1.51	20.55 ± 1.10	20.61 ± 0.98	20.13 ± 1.15
7	20.08 ± 1.32	20.97 ± 1.38	20.15 ± 1.16	20.81 ± 1.31	20.40 ± 0.87
8	20.78 ± 1.23	21.85 ± 1.64	21.11 ± 1.23	21.64 ± 1.05	21.30 ± 1.00
9	21.22 ± 1.36	21.87 ± 1.69	21.72 ± 1.53	21.52 ± 0.91	21.41 ± 0.89

The mean and SD values are shown for the five groups of mice. Each group contains 12 mice.

**Table 4:** Mice spleen weight records.

Group	Control	FL200	FL400	FL800	FL1200
Spleen Weight (g)	0.168 ± 0.028	0.154 ± 0.031	0.147 ± 0.031	0.155 ± 0.038	0.180 ± 0.035

The mean and SD values are shown for the five groups of mice. Each group contains 12 mice.

**Table 5:** The individual subpopulations of immune cells in the splenocyte.

Group	Control	FL200	FL400	FL800	FL1200
	Splenocyte (%)				
MHC Ⅱ	52.7 ± 3.9	54.9 ± 3.6	55.5 ± 3.5	54.6 ± 3.7	52.6 ± 5.6
Total T cell	27.4 ± 3.6	26.0 ± 2.6	26.7 ± 3.4	26.9 ± 2.1	27.7 ± 3.5
Total B cell	41.8 ± 2.5	43.8 ± 4.6	43.0 ± 5.4	42.4 ± 4.0	41.9 ± 5.5
CD4^+^ T cell	20.8 ± 2.5	20.5 ±2.4	20.9 ± 3.2	20.5 ± 1.4	21.3 ± 3.0
CD8^+^ T cell	8.5 ± 1.2	8.1 ± 1.1	8.2 ± 1.4	8.0 ± 0.9	8.3 ± 1.7
NK cell	4.6 ± 0.8	5.2 ± 1.3	4.8 ± 0.9	4.9 ± 0.5	5.0 ± 0.8
Macrophage	14.9 ± 5.9	15.1 ± 5.7	14.4 ± 5.4	14.2 ± 5.0	14.3 ± 4.6

The mean and SD values are shown for the five groups of mice. Each group contains 12 mice.

**Table 6:** The NK cells activity analysis in mice spleen cells. The mean and SD values are shown for the five groups of mice.

Group	Control	FL200	FL400%	FL800	FL1200
NK cells activity	34.1 ± 5.4 ^ab^	33.7 ± 5.5 ^b^	40.6 ± 6.3 ^a^	38.0 ± 6.7 ^ab^	39.1 ± 5.3 ^ab^

Each group contains 12 mice. Data were analyzed statistically using one-way ANOVA and Dunnett’s test. ^a,b^ Values with different superscript letters means significantly different between groups (*p* < 0.05).

**Table 7:** IFN-γ (the Th1 immune response) and IL-5 (the Th2 immune response) concentration (pg/mL) of ConA-induced mice spleen cells isolated from mice treated with FL200, FL400, FL800, and FL1200.

Group	Control	FL200	FL400	FL800	FL1200
	pg/mL				
Th1					
IFN-γ	6807.6 ± 2541.6 ^b^	6677.6 ± 2153.5 ^b^	7551.4 ± 2083.6 ^b^	10126.5 ± 3349.3 ^ab^	11534.3 ± 4907.1 ^a^
Th2					
IL-5	526.2 ± 402.9 ^a^	179.3 ± 154.2 ^b^	77.9 ± 89.2 ^b^	89.6 ± 93.2 ^b^	133.7 ± 92.8 ^b^

The mean and SD values are shown for the five groups of mice. Each group contains 12 mice. Data were analyzed statistically using one-way ANOVA and Dunnett’s test. ^a,b^ Values with different superscript letters mean significant difference between groups (*p* < 0.05).

**Table 8:** IFN-γ (The Th1 immune response) concentration (pg/mL) of LPS-induced mice spleen cells isolated from mice treated with FL200, FL400, FL800, and FL1200.

Group	Control	FL200	FL400	FL800	FL1200
	pg/mL				
IFN-γ	211.1 ± 231.3 ^c^	486.6 ± 434.3 ^b,c^	793.9 ± 546.3 ^a,b^	903.0 ± 449.3 ^a,b^	1055.3 ± 679.5 ^a^

The mean and SD values are shown for the five groups of mice. Each group contains 12 mice. Data were analyzed statistically using one-way ANOVA and Dunnett’s test. ^a,b,c^ Values with different superscript letters mean significant difference between groups (*p* < 0.05).

**Table 9:** IL-4 (Th2 immune response) concentration (pg/mL) of mice spleen cell isolated from OVA-induced mice treated with FL200, FL400, FL800, and FL1200.

Group	Control	FL200	FL400	FL800	FL1200
	pg/mL				
IL-4	21.0 ± 12.9	20.0 ± 19.6	22.2 ± 13.5	11.3 ± 5.8 *	11.8 ± 9.6

The mean and SD values are shown for the five groups of mice. Each group contains 12 mice. Data were analyzed statistically using one-way ANOVA and Dunnett’s test. * *p* < 0.05 compared with control group.

## Data Availability

The data presented in this study are available on request from the corresponding authors. The data are not publicly available.

## Supplementary Materials

The following are available online at https://www.mdpi.com/2075-1729/11/2/111/s1, Figure S1: The UPLC chromatogram of tumulosic acid (**1**), Figure S2: The ESI-MS spectrum of tumulosic acid (**1**), Figure S3: The UPLC chromatogram of polyporenic acid C (**2**), Figure S4: The ESI-MS spectrum of polyporenic acid C (**2**), Figure S5: The UPLC chromatogram of 3-epi-dehydrotumulosic acid (**3**), Figure S6: The ESI-MS spectrum of 3-epi-dehydrotumulosic acid (**3**), Figure S7: The UPLC chromatogram of dehydrotumulosic acid (**4**), Figure S8: The ESI-MS spectrum of dehydrotumulosic acid (**4**), Table S1: 1H NMR (400 MHz) and 13C (100 MHz) Spectroscopic Data for **1**–**4**.
