# Supplementary material for: The Lanostane Triterpenoids in Poria cocos Play Beneficial Roles in Immunoregulatory Activity
Source: Life (Basel). 2021 Feb 1;11(2):111. doi: 10.3390/life11020111 (PMC7912843; doi:10.3390/life11020111)
Supplement: Supplementary file 1 [file life-11-00111-s001.pdf]

The Lanostane Triterpenoids in *Poria cocos* Play Beneficial Roles in Immunoregulatory Activity

Chien-Liang Chao[1], Hsin-Wen Huang[1], Muh-Hwan Su[1, 2], Hang-Ching Lin[1,2,*] and Wen-Mein Wu[3,*]

[1] Sinphar Pharmaceutical Co., Ltd., Sinphar group, Yilan 269, Taiwan;
[2] School of Pharmacy, National Defense Medical Center, Taipei 114, Taiwan;
[3] Department of Nutritional Science, Fu-Jen Catholic University, Hsinchuang 24205, Taiwan;
* Correspondence: lhc@sinphar.com.tw (H.-C.L.); 050582@mail.fju.edu.tw (W.-M.W.); Tel.: +886-2-2905-3633 (W.-M.W.); +886-2-87923100 (ext.18879) (H.-C.L.)

1. The Characteristics of Triterpenoid Compounds (1–4).

Tumulosic Acid (**1**): white powder; ESI−MS m/z: 485.3635 $[M-H]^-$; ^1H NMR (400 MHz, Pyridine-d_5) and ^{13}C-NMR (100 MHz, Pyridine-d_5) data see Table S1.

Polyporenic Acid C (**2**): white powder; ESI−MS m/z: 481.3332 $[M-H]^-$; ^1H NMR (400 MHz, Pyridine-d_5) and ^{13}C-NMR (100 MHz, Pyridine-d_5) data see Table S1.

3-Epi-Dehydrotumulosic Acid (**3**): white powder; ESI−MS m/z: 483.3533 $[M-H]^-$; ^1H NMR (400 MHz, Pyridine-d_5) and ^{13}C-NMR (100 MHz, Pyridine-d_5) data see Table S1.

Dehydrotumulosic Acid (**4**): white powder; ESI−MS m/z: 483.3475 $[M-H]^-$; ^1H NMR (400 MHz, Pyridine-d_5) and ^{13}C-NMR (100 MHz, Pyridine-d_5) data see Table S1.

Table 1. ^1H NMR (400 MHz) and ^{13}C (100 MHz) Spectroscopic Data for **1**-**4**.

	1		2		3		4	
no.	δ_H multi (J in Hz)	δ_C	δ_H multi (J in Hz)	δ_C	δ_H multi (J in Hz)	δ_C	δ_H multi (J in Hz)	δ_C
1	1.65 (br t, J = 13)	36.58 t	1.65 (td, J=14, 4.3); 2.09 (m)	36.8 t	2.26 (m); 1.72(br d, J=12.4)	30.6 t	1.97 (m)；2.57 (m)	36.74 t
2	1.88 (m)；1.96 (m)	29.13 t	2.3; 2.75 (m)	34.9 t	1.88 (dd, J=13.7, 8.4) 2.07 (m)	26.7 t	1.51 (m)；1.95 (m)	29.14 t
3	3.43 (t, J = 8.0)	78.53 d		215.2 s	3.62 (s)	75.1 d	3.48 (t, J=8.0)	78.47 d
4		40.02 s		47.5 s		38.0 s		39.82 s
5	1.18 (d, J = 3.8)	51.42 d	1.59 (dd, J=11.5, 3.7)	51.0 d	2.01 (br t, J=8.3)	43.7 d	1.32 (d, J=3.8)	50.27 d
6	1.56 (m)；1.76 (m)	19.21 t	1.96 (m); 2.13 (m)	23.9 t	2.10 (m); 2.10 (m)	23.4 t	2.16 (m)	23.99 t
7	2.13 (m)	27.47 t	5.57 (br d, J=6)	120.7 d	5.62 (s)	121.3 d	5.65 (br s)	121.76 d
8		135.39 s		142.8 s		142.8 s		143.19 s
9		135.29 s		144.7 s		146.7 s		146.86 s
10		37.88 s		37.5 s		37.9 s		38.34 s
11	2.01 (m)	21.46 t	5.35 (br d, J=5.6)	117.7 d	5.46 (d, J=5.7)	116.2 d	5.40 (br s)	117.02 d
12	2.20 (d, J = 12.5)；2.00 (m)	30.20 t	2.37; 2.67 (br d, J=18.2)	36.3 t	2.64 (br d, J=15.6); 2.40 (m)	36.3 t	2.71 (d, J=12.5)；2.42 (m)	36.79 t
13		49.22 s		45.0 s		45.1 s		45.56 s
14		46.76 s		49.4 s		49.5 s		49.89 s
15	1.74 (d, J=13)；2.42 (dd, J=13, 8)	44.18 t	2.42; 1.91 (d, J=13)	44.4 t	2.40 (m)	44.5 t	1.74 (d, J=13)；2.42 (dd, J=13, 8)	44.92 t
16	4.56 (t, J=6.8)	77.16 d	4.52 (t, J=7, 6.8)	76.4 d	4.51 (t, J=7.2, 6.5)	76.5 d	4.56 (t, J=6.8)	76.91 d
17	2.80 (dd, J=11.1, 5.9)	57.79 d	2.86 (dd, J=11.2, 5.8)	57.6 d	2.85 (dd, J=11.1, 5.7)	57.6 d	2.92 (dd, J=11.1, 5.9)	58.12 d
18	1.17 (s)	18.28 q	1.04 (s)	17.6 q	1.08 (s)	17.7 q	1.09 (s)	18.11 q
19	1.05 (s)	19.42 q	1.12 (s)	22.0 q	1.09 (s)	23.0 q	1.09 (s)	23.47 q
20	2.93 (m)	49.28 d	2.94 (m)	48.5 d	2.94 (br t, J=9.5)	48.6 d	2.92 (m)	49.03 d
21		179.49 s		178.7 s		178.7 s		177.87 s
22	2.66 (m)；2.50 (m)	32.08 t	2.46 (m); 2.62 (m)	31.4 t	2.45 (m); 2.70 (m)	31.5 t	2.51 (m)；2.43 (m)	31.94 t
23	2.41 (br t, J=12)；2.57 (br t, J=12)	33.72 t	2.41 (m); 2.53 (m)	33.2 t	2.37; 2.52 (t, J=11.6)	33.2 t	2.41 (br t, J=12)；2.53 (br t, J=12)	33.69 t
24		156.58 s		156.0 s		156.1 s		156.52 s

25	2.30 (m)	34.61 d	2.25 (m)	34.1 d	2.26 (m)	34.1 d	2.33 (m)	34.58 d
26	0.97 (d, J=6.75)	22.52 q	0.97 (dd, J=7.2)	22.0 q	0.96 (d, J=7)	22.0 q	1.01 (d, J=6.75)	22.48 q
27	0.98 (d, J=6.75)	22.38 q	0.99 (dd, J=7.2)	21.9 q	0.98 (d, J=7)	21.9 q	1.01 (d, J=6.75)	22.34 q
28	1.23 (s)	29. 15 q	1.12 (s)	25.7 q	1.18 (s)	29.2 q	1.23 (s)	29.31 q
29	1.06 (s)	16.87 q	1.04 (s)	22.4 q	0.98 (s)	23.2 q	1.15 (s)	17.12 q
30	1.47 (s)	25.94 q	1.44 (s)	26.4 q	1.42 (s)	26.6 q	1.53 (s)	27.08 q
31	4.83 (s)；4.97 (s)	107.01 t	4.83 (s)；4.97 (s)	107.5 t	4.83 (s); 5.02 (s)	107.1 t	4.86 (s)；5.00 (s)	107.51 t

2. Chromatogram of Triterpenoid Compounds (1-4) using UPLC/MS.

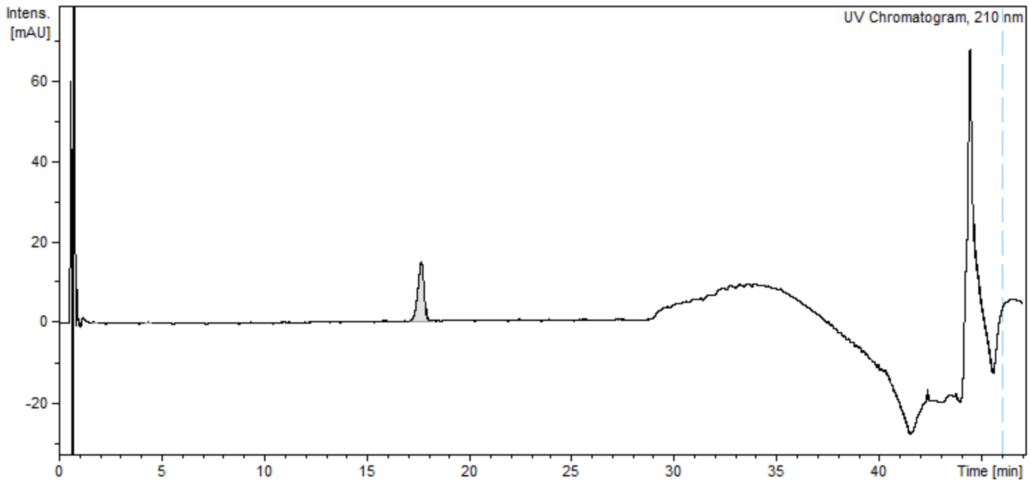

Figure S1. The UPLC chromatogram of tumulosic acid (**1**).

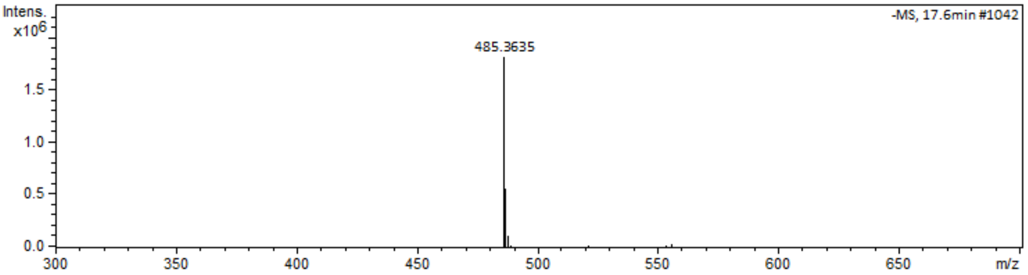

Figure S2. The ESI-MS spectrum of tumulosic acid (**1**).

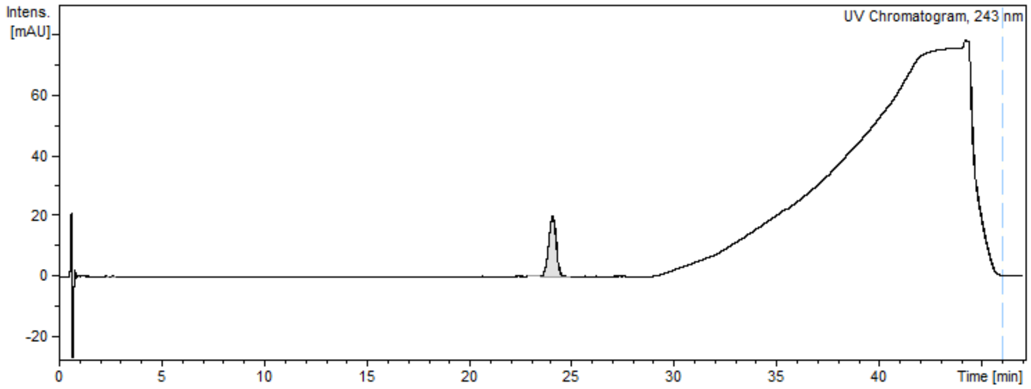

Figure S3. The UPLC chromatogram of polyporenic acid C (**2**).

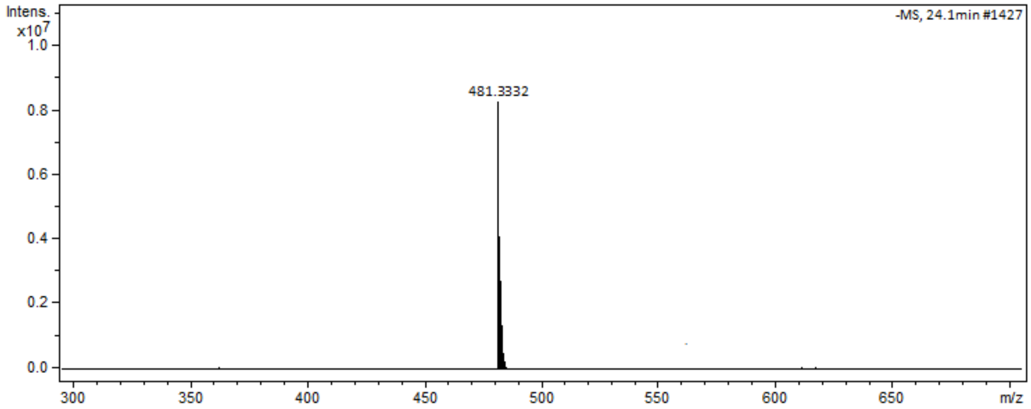

Figure S4. The ESI-MS spectrum of polyporenic acid C (**2**).

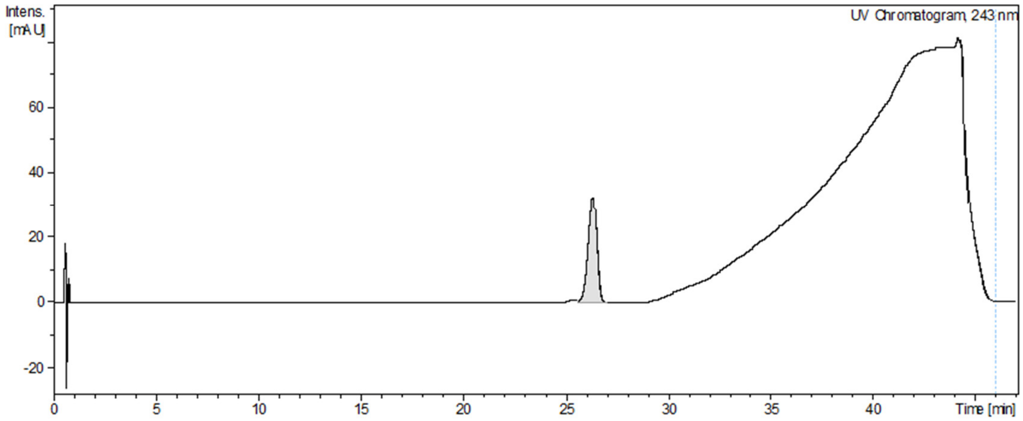

Figure S5. The UPLC chromatogram of 3-epi-dehydrotumulosic acid (**3**).

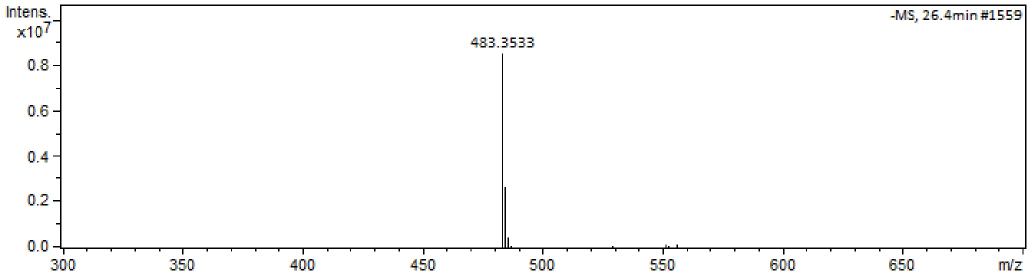

Figure S6. The ESI-MS spectrum of 3-epi-dehydrotumulosic acid (**3**).

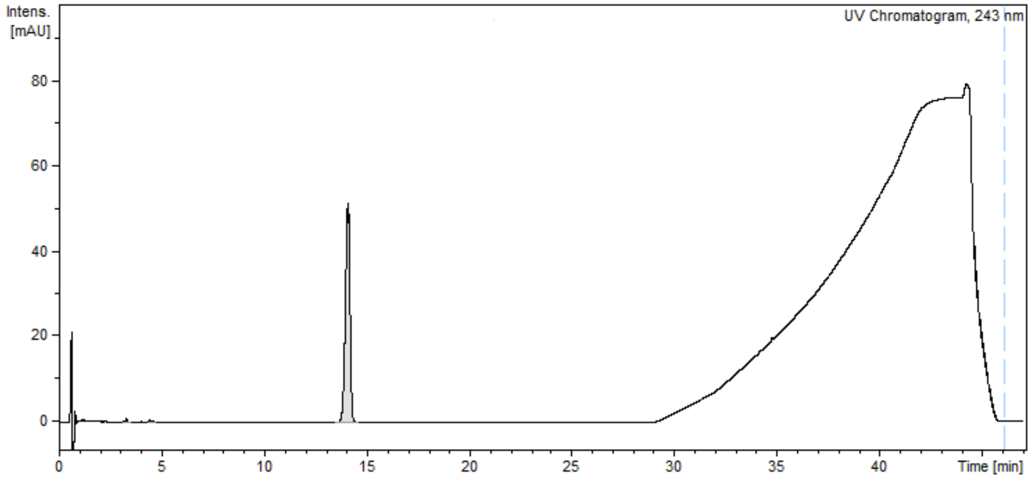

Figure S7. The UPLC chromatogram of dehydrotumulosic acid (**4**).

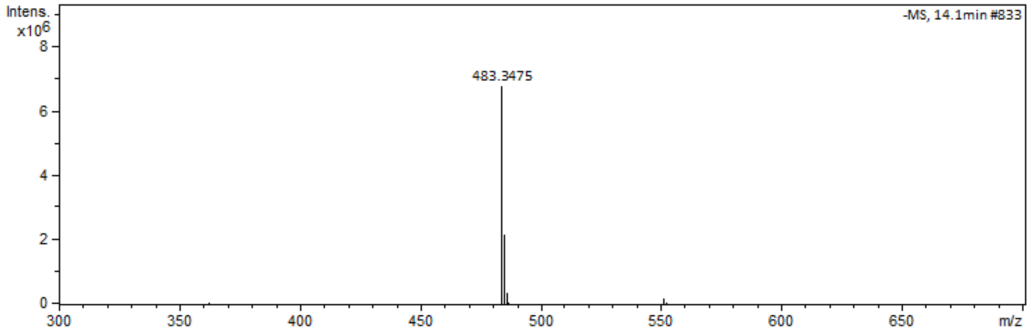

Figure S8. The ESI-MS spectrum of dehydrotumulosic acid (**4**).
